# Resting-State Functional Connectivity of the Punishment Network Associated With Conformity

**DOI:** 10.3389/fnbeh.2020.617402

**Published:** 2020-12-16

**Authors:** Yin Du, Yinan Wang, Mengxia Yu, Xue Tian, Jia Liu

**Affiliations:** ^1^Faculty of Psychology, Beijing Normal University, Beijing, China; ^2^Department of Psychology, Tsinghua Laboratory of Brain and Intelligence, Tsinghua University, Beijing, China

**Keywords:** punishment network, functional connectivity, conformity propensity, thalamus, insula, postcentral gyrus, dACC

## Abstract

Fear of punishment prompts individuals to conform. However, why some people are more inclined than others to conform despite being unaware of any obvious punishment remains unclear, which means the dispositional determinants of individual differences in conformity propensity are poorly understood. Here, we explored whether such individual differences might be explained by individuals’ stable neural markers to potential punishment. To do this, we first defined the punishment network (PN) by combining all potential brain regions involved in punishment processing. We subsequently used a voxel-based global brain connectivity (GBC) method based on resting-state functional connectivity (FC) to characterize the hubs in the PN, which reflected an ongoing readiness state (i.e., sensitivity) for potential punishment. Then, we used the within-network connectivity (WNC) of each voxel in the PN of 264 participants to explain their tendency to conform by using a conformity scale. We found that a stronger WNC in the right thalamus, left insula, postcentral gyrus, and dACC was associated with a stronger tendency to conform. Furthermore, the FC among the four hubs seemed to form a three-phase ascending pathway, contributing to conformity propensity at every phase. Thus, our results suggest that task-independent spontaneous connectivity in the PN could predispose individuals to conform.

## Introduction

“*The idea that men are created free and equal is both true and misleading: men are created different; they lose their freedom and their autonomy in seeking to become like each other*.”

David Riesman, *The Lonely Crowd: A Study of the Changing American Character*

Conformity is a prevailing social phenomenon, which means behaving in accordance with the common norms, social standards, attitudes, beliefs, and values of a given culture (Riesman, 1950; Riesman et al., 2001). At an individual level, conformity refers to the act of changing one’s behavior to match the responses of others (Cialdini and Goldstein, 2004). Marcuse (1964) defined this social character as one-dimensionality in his book, *One-Dimensional Man*, describing a state of affairs that conforms to existing thought and behavior, in which there is a lack of critical dimension.

Individuals are prompted to conform due to a fear of punishment (Cialdini and Goldstein, 2004; Spitzer et al., 2007; Haun and Tomasello, 2011; Gelfand, 2012). First, from a social psychological perspective, a minority position is aversive (Asch, 1956; Hornsey et al., 2003); it can lead to hostility, disapproval, rejection from others, or social isolation (Heerdink et al., 2015). To avoid such social punishment, people might be motivated to conform to the majority position (Falk et al., 2012). Second, from an evolutionary perspective, evolutionary game-theoretic models (Smith, 1982) show that groups that face greater societal threats require harsher punishment for norm deviators to avoid a breakdown of cooperation and to survive (Gelfand, 2012). Regarding the prominent role of such peer punishment in human evolution (Boyd et al., 2003), humans could have developed corresponding neural mechanisms that made them constantly vigilant to the threat of potential punishment (Fehr and Gächter, 2002; De Quervain et al., 2004; Spitzer et al., 2007), which implies that the dispositional determinants of individual differences in conformity propensity (Egerton et al., 2010; Jolles et al., 2011) might be a stable neural trait (i.e., sensitivity to punishment at a neural level). Also, as the tendency to imitate is usually swift and automatic (Griskevicius et al., 2006), the individual differences in conformity propensity might be driven by differences in early automatic perception of potential punishment (Franzen and Brinkmann, 2015).

Therefore, in the present study, we explored whether and what neural traits—dispositional brain-based characteristics—might explain individual differences in conformity propensity. To measure individuals’ neural markers of punishment sensitivity, one of the best options is to use spontaneous resting-state functional magnetic resonance imaging (rs-fMRI) to measure the level of coactivation of functional time series [i.e., functional connectivity (FC)] in a specific functional network [i.e., the brain punishment network (PN; Salvador et al., 2005; Damoiseaux et al., 2006; Van den Heuvel and Hulshoff Pol, 2010)]. Because brain regions often have to work together to form a functional network during rest (Damoiseaux et al., 2006; Fox and Raichle, 2007; Smith et al., 2013), this makes spontaneous rs-fMRI oscillations a robust measure to examine ongoing functional communication between brain regions absent of actual stimulus (Peelen et al., 2013; Hutchison et al., 2014; Stevens et al., 2015; Wang et al., 2016). Unlike task-based imaging, which typically highlights brain responses associated with any given task, rs-fMRI allows researchers to observe how a brain’s resting-state connectivity is ready for prime time in the absence of any explicit task (Shen, 2015). Therefore, we can measure rs-fMRI in PN to characterize individuals’ preparation and anticipation states for potential punishment. Hence, the resting-state FC in the PN is an ideal neural marker of punishment sensitivity.

Here, we defined the PN by including all brain regions potentially involved in punishment processing. According to neuroscience studies, punishment processing may be underpinned by several distinct brain systems (Palminteri and Pessiglione, 2017). The first system has suggested that punishment-avoidance processing is driven by dopamine (DA) activity (Brooks and Berns, 2013). Specifically, some fMRI studies have shown that the dorsal parts of the frontostriatal circuits (dorsal striatum) could reinforce punishment avoidance (Seymour et al., 2007; Delgado et al., 2008; Shenhav and Buckner, 2014; Pauli et al., 2015). Additionally, some studies have emphasized that punishment processing is mediated by aversive signals encoded in other brain areas, such as the insula, dorsal anterior cingulate cortex (dACC), and amygdala (Gonzalez et al., 2014; Namburi et al., 2016; Bernardi and Salzman, 2017). The involvement of these regions in experiencing punishment has been supported by some fMRI studies as well as meta-analyses (Palminteri et al., 2012, 2015; Bartra et al., 2013; Garrison et al., 2013; Hayes et al., 2014). These results demonstrate the critical and specific role that various brain structures could play in punishment sensitivity: first, some were implicated in the DA system (striatum), and second, other subcortical and cortical structures were implicated in aversive processing, such as the insula, dACC, and amygdala. Therefore, the aforementioned brain regions all possibly contributed to punishment sensitivity and worked in an integrative manner, despite the absence of any punishment stimulus, to predispose individuals to conform.

To test this hypothesis, we first combined all potential brain regions associated with punishment processing to form the PN using an automated meta-analysis (i.e., Neurosynth; Palminteri and Pessiglione, 2017). Then, we characterized the voxel-wise FC within the PN in a large sample of participants (*N* = 272) with voxel-based global brain connectivity (GBC) method using rs-fMRI (Cole et al., 2012; Wang et al., 2016). For the brain-wide GBC analyses, a voxel’s GBC was computed as the average connectivity of that voxel with the rest of the brain; For the ROI GBC analyses, voxel-wise connectivity was based on average correlations of a voxel with the rest of all within-region voxels (Cole et al., 2010). Thus, in this study, to focus on investigating punishment sensitivity the voxel-wise GBC maps were computed within the PN. Specifically, the functional integration of the PN was determined by calculating the within-network connectivity (WNC) of each voxel in the PN as the average FC of a voxel with the rest of the punishment-selective voxels in the PN. Next, we examined whether the WNC in the PN was related to conformity propensity (tendency), measured with a conformity scale (Mehrabian and Stefl, 1995) to explore attributes of conforming. Hence, by correlating the WNC of each voxel in the PN with the tendency of conformity across participants, we characterized the conformity propensity relevance of integration (i.e., a stronger WNC) of the PN, which could elucidate the dispositional determinant (i.e., punishment sensitivity) of conformity behaviors. We hypothesized that the integration (i.e., a stronger WNC) of the PN is positively associated with conformity propensity.

## Materials and Methods

### Participants

A total of 272 participants [146 female participants; 272 self-reported right-handed; mean age = 20.4 years, standard deviation (SD) = 0.9 years] from Beijing Normal University participated in the rs-fMRI scan and behavioral session. All participants had a normal or corrected-to-normal vision and reported no history of neurological or psychiatric disorders (Table 1). All investigation protocols were approved by the Institutional Review Board of Beijing Normal University. Written informed consent was obtained from all the participants before the study.

**Table 1:** Demographic information for participants.

	*n* = 272
Age	20.4 (0.9)
Gender	146 F (54%), 126 M (46%)
Left-handed	0%
History of neurological disorders	N/A
History of psychiatric disorders	N/A

### PN Map From Neurosynth Meta-analysis

To obtain an activation map relevant for punishment processing, we used an automated meta-analysis tool called Neurosynth^1^ (Yarkoni et al., 2011) to generate the association test map displaying brain regions preferentially related to the key terms “punishment,” “aversive,” and “pain” (Palminteri and Pessiglione, 2017). The meta-analysis was performed by automatically identifying all studies in the Neurosynth database that loaded highly on the term. Meta-analyses were then performed to identify brain regions consistently or preferentially reported in the tables of those studies, including the key terms. Despite the automaticity and potentially high noise resulting from the association between the term frequency and coordinate tables, this approach has been demonstrated to be robust and reliable (Yarkoni et al., 2011; Helfinstein et al., 2014; Kong et al., 2017). The database was accessed in February 2019. “Punishment” was searched for in 92 studies with 2,881 activations, “aversive” was searched for in 238 studies with 8,529 activations, and “pain” was searched for in 516 studies with 23,295 activations. The generated maps were corrected using a false discovery rate (FDR) approach with an expected FDR of 0.01. We combined all three maps to create the final map of the PN. As expected, the resulting statistical map included the dACC, postcentral gyrus (PG), bilateral insula, striatum, thalamus, and amygdala, which is similar to the results obtained in previous punishment-processing studies (Delgado et al., 2008; Palminteri et al., 2012; Bartra et al., 2013; Garrison et al., 2013; Eisenberger, 2015; Pauli et al., 2015; Bernardi and Salzman, 2017; Palminteri and Pessiglione, 2017).

### Image Acquisition

The images were acquired using a 3T scanner (MAGNETOM Trio, A Tim System; Siemens) with a 12-channel phased-array head coil at the Beijing Normal University Imaging Center for Brain Research in Beijing, China. The rs-fMRI scanning was conducted using a gradient-echo echo-planar imaging (GRE-EPI) sequence [repetition time (TR) = 2,000 ms, echo time (TE) = 30 ms, flip angle = 90°, number of slices = 33, voxel size = 3.125 × 3.125 × 3.6 mm^3^]. Scanning lasted for 8 min and consisted of 240 contiguous EPI volumes. During the scan, the participants were instructed to relax without engaging in any specific task and remain still with their eyes closed. In addition, high-resolution T1-weighted images were acquired with a magnetization-prepared gradient-echo sequence (MPRAGE: TR/TE/TI = 2,530/3.39/1,100 ms, flip angle = 7°, matrix = 256 × 256, number of slices = 128, and voxel size = 1 × 1 × 1.33 mm^3^) for spatial registration. Earplugs were used to attenuate scanner noise, and a foam pillow and extendable padded head clamps were used to restrain the participants’ head motion.

### Image Preprocessing

The rs-fMRI data were preprocessed using the FMRIB Software Library (FSL)^2^. Preprocessing included removal of the first four images, correction for head motion (by aligning each volume to the middle volume of the image with the MCFLIRT), spatial Gaussian smoothing [with a Gaussian kernel of 6 mm full-width at half-maximum (FWHM)], intensity normalization, and linear trend removal. A temporal bandpass filter (0.01–0.1 Hz) was then applied to reduce low-frequency drifts and high-frequency noise.

To further eliminate physiological noise, such as the fluctuations caused by motion, cardiac and respiratory cycles, nuisance signals from cerebrospinal fluid, white matter, whole-brain average, motion correction parameters, and the first derivatives of these signals were regressed out using the methods described in previous studies (Fox et al., 2005; Biswal et al., 2010). The four-dimensional residual time series obtained after removing the nuisance covariates were used for the rs-FC analyses. The strength of the intrinsic FC between two voxels was estimated using Pearson’s correlation of the residual resting-state time series for those voxels.

The rs-fMRI images of each participant to the structural images were registered using FLIRT to produce a six-degrees-of-freedom affine transformation matrix. The registration of each participant’s structural images to a common stereotaxic space [the Montreal Neurological Institute (MNI) 152-brain template with a resolution of 2 × 2 × 2 mm^3^, MNI152] was accomplished using FLIRT to produce a 12-degrees-of-freedom linear affine matrix (Jenkinson and Smith, 2001; Jenkinson et al., 2002).

### Behavioral Tests

The participants’ conformity propensity was measured using an 11-items conformity scale based on Mehrabian and Stefl (1995). Conformity was defined as involving the characteristic willingness to identify with others and emulate them, giving in to others to avoid conflict, and being a follower rather than a leader in terms of ideas, values, and behaviors (Mehrabian and Stefl, 1995). Seven items were positively scored (+), showing a stronger tendency toward conformity, while the remaining four items were negatively scored (−). The items are statements such as “I often rely and act upon the advice of others” (+), “Generally, I’d rather give in and go along with the majority of others for consistency” (+), and “I am more independent than conforming in my ways” (−). The participants were asked to evaluate themselves on a 6-point Likert scale ranging from 1 (never or almost never true) to 6 (always or almost always true), with higher scores indicating a higher tendency to conform. In the current study, the internal consistency for all items was provided by a Cronbach’s coefficient of 0.78.

### WNC Analyses in the PN

The GBC method, which is a recently developed analytical approach for fMRI data, was used to characterize the intrinsic WNC of each voxel within the PN (Cole et al., 2012). The GBC of a voxel was generally defined as the averaged FC of that voxel to the remaining voxels in the entire brain or a predefined mask (Cole et al., 2012; Wang et al., 2016; Pan et al., 2019; Li et al., 2020). This method enabled the characterization of a specific region’s full-range FC with the voxel-wise resolution, allowing us to comprehensively examine the role of each region’s FC in punishment sensitivity. Specifically, the FC of a PN voxel to the remaining PN voxels was computed one by one and then averaged as the WNC of the PN voxel. Then, participant-level WNC maps were transformed to *z*-score maps by using Fisher’s *z-transformation* to yield normally distributed values (Cole et al., 2012; Gotts et al., 2013). A one-sample *t*-test was performed for each voxel WNC to identify the distribution of hub regions within the PN (Song et al., 2020). The significance was determined using the FDR correction approach with *p* < 0.01. Moreover, we conducted two-sample *t*-tests to compare the WNC in the PN between male and female participants to determine whether gender differences existed in punishment sensitivity. The significance was determined using the FDR correction approach with *p* < 0.01.

### WNC–Conformity Propensity Correlation Analyses

A correlation analysis was conducted to examine the relationship between the WNC of each voxel in the PN and the individual differences in conformity propensity. Specifically, a Pearson’s correlation between the WNC and conformity scores was conducted for each voxel with a GLM tool implemented in FSL, where the conformity scores were set as an independent variable and the WNC in the PN was set as the dependent variable. Multiple comparison correction was performed on the statistical map using the 3dClustSim program implemented in AFNI^3^ (version 16.1.13, 2016). The voxel- and cluster-level thresholds of *p* < 0.002 and *p* < 0.05, respectively, were set based on Monte Carlo simulations in the PN mask.

Furthermore, control analyses were performed to rule out other possible confounding factors such as head motion and gender, because recent studies have shown that rs-FC is largely affected by head motion (Satterthwaite et al., 2012; Van Dijk et al., 2012) and gender was identified as a possible modulator of conformity (Rosander and Eriksson, 2012). Thus, we calculated the partial correlation between WNC and conformity propensity while controlling for head motion and gender. The extent of head motion was measured by the mean framewise displacement (FD) for each participant (Van Dijk et al., 2012).

### Seed-Based FC-Conformity Correlation Analysis

We further investigated with which specific regions the FCs of the identified clusters in the aforementioned WNC–conformity correlation analyses were correlated with conformity propensity. In this regard, seed-based FC analyses were performed with each identified cluster as the seed. For a seed identified in the WNC–conformity correlation analysis, we calculated the FC between the mean time series in the seed (Fisher’s *z*-transformed) and each PN voxel and correlated the FC with conformity scores. Again, multiple comparison correction was performed using the 3dClusSim program implemented in AFNI (version 16.1.13, 2016)^3^. A threshold of voxel-level *p* < 0.002 and cluster-level *p* < 0.05 was set based on Monte Carlo simulations in the PN. Furthermore, similar control analyses were performed to rule out the confounding effects of head motion and gender.

### Participant Exclusion

The exclusion criterion for fMRI data was head motion >2.0° in rotation or 2.0 mm in translation throughout the fMRI scan. Four participants (three male and one female) were excluded based on this criterion. For the behavioral tests, Tukey’s outlier filter (Hoaglin et al., 1983) was used to identify outlier participants with exceptionally low (3 × the interquartile range below the first quartile) or high (3 × the interquartile range above the third quartile) scores. Four additional participants (two male and two female) were excluded using this method.

## Results

### Behavior Results

Participants’ conformity propensity was measured using the conformity scale (Mehrabian and Stefl, 1995), and the mean score obtained by the sample (*N* = 264) was 3.47 (*SD* = 0.58). Also, an independent sample *t*-test was used to examine the difference in the conformity propensity between male and female participants. The results revealed significant differences between male (mean = 3.29, *SD* = 0.56) and female (mean = 3.63, *SD* = 0.55), *t*_(262)_ = 5.06, *p* < 0.001, Cohen’s *d* = 0.61) participants, which is consistent with previous studies that a gender difference in conformity propensity might exist at the behavioral level (Rosander and Eriksson, 2012). Therefore, we used gender as a control variable for further analysis.

### Definition of PN

**Figure 1:**
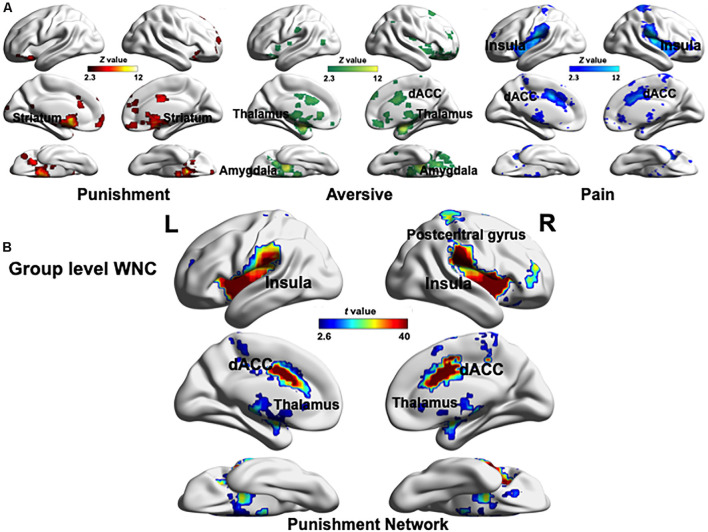
**(A)** Maps resulting from automatized large-scale meta-analyses as implemented in Neurosynth. Three association test maps displaying brain regions preferentially related to the key terms “punishment,” “aversive,” and “pain” created in the Neurosynth meta-analysis (*Z* > 2.3, uncorrected). These three maps involve both similar (the dACC) and specific (notably the striatum, thalamus, amygdala, and insula) brain regions. **(B)** Global pattern of within-network connectivity (WNC) in the punishment network (PN; combing aforementioned “punishment,” “aversive,” and “pain” maps). The group-level (one-sample *t*-test) WNC map is overlaid on the cortical surface (FDR corrected *p* < 0.01). L, left; R, right. The visualization was provided by BrainNet Viewer (http://www.nitrc.org/projects/bnv/).

To define the PN, we used the results of the Neurosynth meta-analysis with the terms “punishment,” “aversive,” and “pain” (*Z* > 2.3, uncorrected, Figure 1A) and recreated a PN mask combining the three association test maps (Palminteri and Pessiglione, 2017). As a result, the PN included the dACC, PG, bilateral insula, thalamus, amygdala, and striatum (Figure 1B). The regions in the PN were in agreement with the punishment-selective regions identified in studies on punishment processing (Delgado et al., 2008; Palminteri et al., 2012; Bartra et al., 2013; Garrison et al., 2013; Eisenberger, 2015; Pauli et al., 2015; Bernardi and Salzman, 2017; Palminteri and Pessiglione, 2017).

### WNC in the PN

After identifying the PN, we computed each voxel’s WNC in the PN by using the rs-fMRI data, where the WNC measured the voxel-wise FC within the PN. First, a one-sample *t*-test was used to identify the hubs distribution in the PN. Specifically, we used a one-sample *t*-test to calculate the WNC across voxels in the entire sample (*N* = 264). The results showed that almost all voxels in the PN exhibited positive WNC (FDR-corrected *p* < 0.01), suggesting that the PN is a relatively encapsulated network, and among all the PN regions (FDR-corrected *p* < 0.01), the insula, thalamus, dACC, and PG had the largest WNC values (Figure 1B), among which the WNC values of the right thalamus, bilateral insula, dACC, and PG was 1 SD higher than the mean WNC value of the PN, suggesting that these regions serve as hubs of the PN (Dai et al., 2015; Wang et al., 2016). Also, a two-sample *t*-test between male and female participants across voxels in the WNC value within the PN revealed no significant difference between genders, which indicated that male and female participants have similar sensitivity to punishment at the neural level.

### Correlation Between WNC and Conformity Propensity

**Figure 2:**
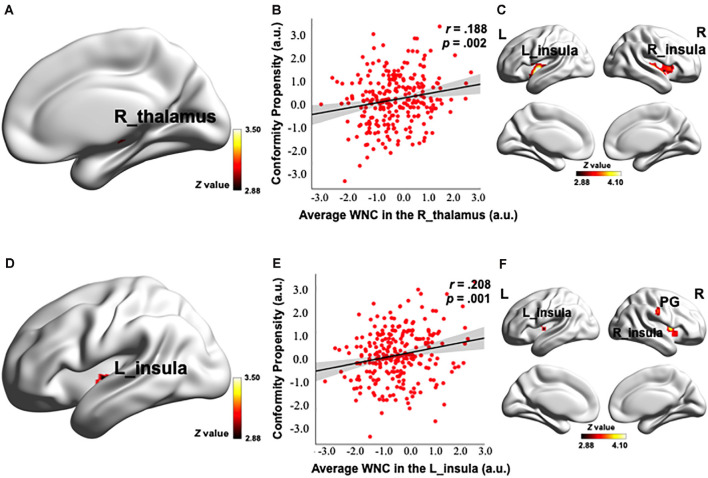
Correlation between the PN WNC and conformity. The PN WNC in the R_thalamus (38 voxels, voxel-level *p* < 0.002, cluster-level *p* < 0.05, corrected) and L_insula (44 voxels, voxel-level *p* < 0.002, cluster-level *p* < 0.05, corrected) was positively correlated with conformity **(A,D)**. The scatter plots are shown just for illustration and visualization of the partial correlation (controlling for gender and head motion) between the PN WNC in the R_thalamus, L_insula, and conformity **(B,E)**. Correlation between seed-based functional connectivity (FC) and conformity is shown in Panels **(C,F)**. **(C)** The FC between the R_thalamus seed and the clusters in the bilateral insula was positively correlated with conformity. **(F)** The FC between the L_insula seed and the clusters in the bilateral insula and postcentral gyrus (PG) was positively correlated with conformity. To better visualize the location of the significant clusters, the boundary of the clusters are shown with a red contour. L, left; R, right; a.u., arbitrary units. The visualization was provided by BrainNet Viewer (http://www.nitrc.org/projects/bnv/).

**Figure 3:**
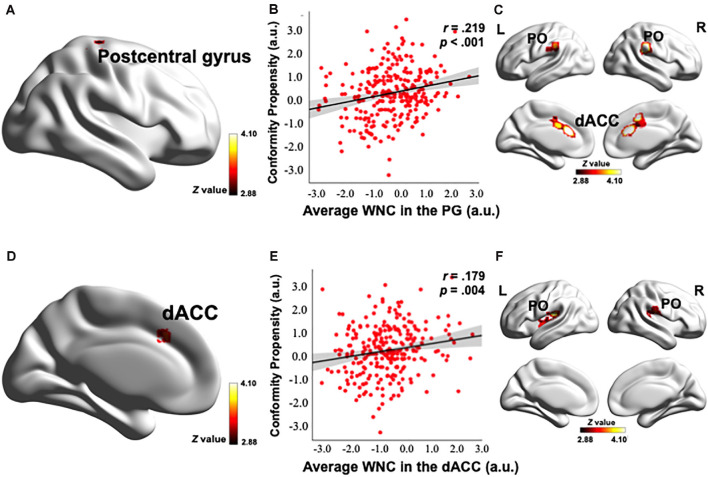
Correlation between the PN WNC and conformity. The PN WNC in the postcentral gyrus (121 voxels, voxel-level *p* < 0.002, cluster-level *p* < 0.05, corrected) and dACC (47 voxels, voxel-level *p* < 0.002, cluster-level *p* < 0.05, corrected) was positively correlated with conformity **(A,D)**. The scatter plots are shown just for illustration and visualization of the partial correlation (controlling for gender and head motion) between the PN WNC in the postcentral gyrus (PG), dACC, and conformity **(B,E)**. Correlation between the seed-based FC and conformity is shown in panels **(C,F)**. **(C)** The FC between the postcentral gyrus seed and the clusters in the dACC and bilateral parietal operculum (PO) was positively correlated with conformity. **(F)** The FC between the dACC seed and the clusters in the bilateral PO was positively correlated with conformity. To better visualize the location of the significant clusters, the boundary of the clusters are shown with a red contour. L, left; R, right; a.u., arbitrary units. The visualization was provided by BrainNet Viewer (http://www.nitrc.org/projects/bnv/).

To investigate how the resting-state FC patterns in the PN were related to conformity propensity, we performed a voxel-wise correlation analysis to search for any PN voxels exhibiting a correlation between WNC and conformity propensity across the participants. As shown in Figures 2A,D, 3A,D and Table 2, four clusters (voxel-level *p* < 0.002, cluster-level *p* < 0.05, corrected) in the right thalamus (38 voxels, *r* = 0.217, *p* < 0.001, MNI coordinates of peak: 14, −22, −2, Figures 2A,B), left insula (44 voxels, *r* = 0.215, *p* < 0.001, MNI coordinates of peak: −38, −10, −4, Figures 2D,E), PG (121 voxels, *r* = 0.252, *p* < 0.001, MNI coordinates of peak: 26, −28, 58, Figures 3A,B), and dACC (47 voxels, *r* = 0.217, *p* < 0.001, MNI coordinates of peak: −42, 24, 34, Figures 3D,E) showed significant positive correlation between the WNC and conformity propensity, suggesting that individuals with stronger within-network integration in these four regions during resting state were more inclined to conform. No clusters showed a negative correlation between the WNC and conformity propensity. In brief, these results suggested that individuals’ conformity propensity was positively correlated with the integration of the right thalamus, left insula, PG, and dACC in the PN.

**Table 2:** Correlation coefficients between conformity propensity and two kinds of functional connectivity (FC) measurements in the punishment network (i.e., voxel-wise WNC and seed-based FC).

WNC
Significant conformity-WNC		Seed-based FC					
correlated brain regions		Conformity seed-based FC correlated brain regions					
Region as seeds	*r*	R_insula	L_insula	PG	R_PO	L_PO	dACC
R_thalamus	0.217*** (0.188**)	0.279*** (0.270***)	0.275*** (0.264***)	-	-	-	-
L_insula	0.215*** (0.208**)	0.255*** (0.254***)	0.248*** (0.239**)	0.207** (0.178**)	-	-	-
PG	0.252*** (0.219***)	-	-	-	0.252*** (0.216***)	0.240*** (0.215***)	0.283*** (0.251***)
dACC	0.217*** (0.179**)	-	-	-	0.231*** (0.210**)	0.290*** (0.262***)	-

Notes: ***correlation is significant at the 0.001 level (two-tailed); **correlation is significant at the 0.01 level (two-tailed). The coefficients of partial correlation controlling for gender and head motion presenting in parentheses. WNC, within-network connectivity; PG, postcentral gyrus; PO, parietal operculum; L, left; R, right.

Control analyses were then performed to ensure that the WNC-conformity correlation in the right thalamus, left insula, PG, and dACC was not caused by confounding factors, such as head motion or gender. We reanalyzed the WNC-conformity correlation while controlling for head motion (Van Dijk et al., 2012) and gender. We found that the correlation remained significant (right thalamus: partial *r* = 0.188, *p* = 0.002; left insula: partial *r* = 0.208, *p* = 0.001; PG: partial *r* = 0.219, *p* < 0.001; dACC: partial *r* = 0.179, *p* = 0.004). These results indicated that the WNC-conformity correlations in the four clusters were not an artifact resulted from head motion or gender.

To examine the reliability of the correlation, the top and bottom 25% of the participants, according to the WNC (in the significantly positive WNC–conformity correlation clusters: right thalamus, left insula, PG and dACC), were labeled as the high- and low-punishment sensitivity groups (*N* = 66 for both groups), respectively. Consistent with the correlation results, the high punishment sensitivity group (a group divided according to the WNC in the cluster in the right thalamus: conformity = 3.60; in the left insula: conformity = 3.58; in the PG: conformity = 3.56; and in the dACC: conformity = 3.64) exhibited higher conformity scores than did the low-punishment sensitivity group (group divided according to WNC in the cluster in the right thalamus: conformity = 3.28; in the left insula: conformity = 3.27; in the PG: conformity = 3.24; and in the dACC: conformity = 3.31) according to the WNC in four clusters, respectively (the difference of conformity scores between the top and low WNC groups in the right thalamus: *t*_(130)_ = −3.099, *p* = 0.002, Cohen’s *d* = 0.55; in the left insula: *t*_(130)_ = −2.946, *p* = 0.004, Cohen’s *d* = 0.51; in the PG: *t*_(130)_ = −3.595, *p* < 0.001, Cohen’s *d* = 0.64; and in the dACC: *t*_(130)_ = −3.351, *p* = 0.001, Cohen’s *d* = 0.58). These results indicated that individuals with superior punishment sensitivity (specifically reflected in the right thalamus, left insula, PG, and dACC integration) are more likely to conform.

### Conformity Relevance of Seed-Based FC in the PN

**Figure 4:**
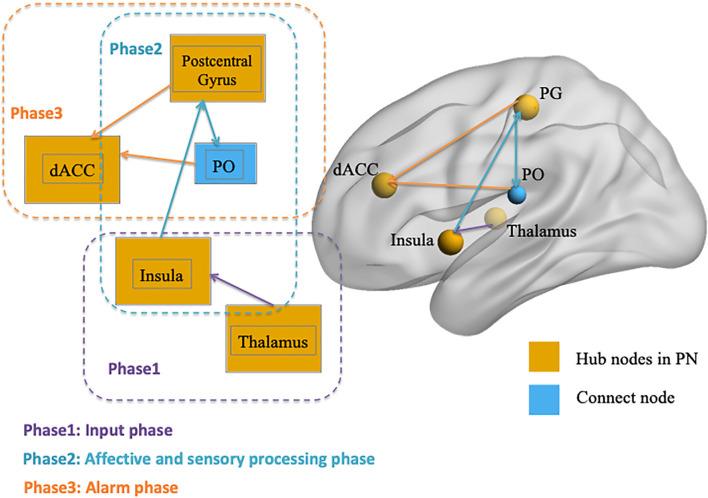
Illustration of the three-phase ascending pathway in the punishment network (PN). Phase 1 is the “input phase” and refers to the connectivity from the thalamus to the insula; Phase 2 is the “affective and sensory processing phase” and refers to the connection between the insula, postcentral gyrus (PG), and parietal operculum (PO); and Phase 3 is the “alarm phase” and refers to the connectivity within PG, PO, and dACC. The visualization was provided by BrainNet Viewer (http://www.nitrc.org/projects/bnv/).

After identifying the right thalamus, left insula, PG, and dACC as the connection hubs within the PN associated with conformity propensity, we examined with which specific regions in the PN the FC of the identified clusters in the aforementioned WNC–conformity correlation analysis were correlated with conformity. For this purpose, we performed seed-based FC analyses with the identified four clusters as seeds. We then calculated the FC between the mean time series in the seed (Fisher’s *z*-transformed) and each PN voxel and correlated the FC with conformity scores (the results of correlation analyses were summarized in Table 2). First, we found that the FC between the right thalamus and two clusters were positively correlated with conformity propensity (voxel-level *p* < 0.002, cluster-level *p* < 0.05, corrected, Figure 2C), including the bilateral insula (right, 655 voxels, MNI coordinates: 40, −11, −4; left, 407 voxels, MNI coordinates: −40, −5, −4). In addition, the correlations remained unchanged while controlling for the participants’ head motion (Van Dijk et al., 2012) and gender (right thalamus-right insula: partial *r* = 0.270, *p* < 0.001; right thalamus-left insula: partial *r* = 0.264, *p* < 0.001). Second, the FC between the left insula and three clusters were positively correlated with conformity propensity (voxel-level *p* < 0.002, cluster-level *p* < 0.05, corrected, Figure 2F), including the PG (59 voxels, MNI coordinates: 60, −15, 34) and the bilateral insula (right, 119 voxels, MNI coordinates: 34, 4, 6; left, 70 voxels, MNI coordinates: −30, −17, 4). Additionally, the correlations remained unchanged while controlling for the participants’ head motion and gender (left insula-PG: partial *r* = 0.178, *p* = 0.004; left insula-right insula: partial *r* = 0.254, *p* < 0.001; left insula-left insula: partial *r* = 0.239, *p* < 0.001). Third, the FC between the PG and three clusters were positively correlated with conformity propensity (voxel-level *p* < 0.002, cluster-level *p* < 0.05, corrected, Figure 3C), including the dACC (759 voxels, MNI coordinates: 8, 24, 20) and the bilateral parietal operculum (PO; right, 334 voxels, MNI coordinates: 56, −29, 32; left, 161 voxels, MNI coordinates: −52, −35, 36). The correlations remained unchanged while controlling for the participants’ head motion and gender (PG-dACC: partial *r* = 0.251, *p* < 0.001; PG-rPO: partial *r* = 0.216, *p* < 0.001; PG-lPO: partial *r* = 0.215, *p* < 0.001). Finally, the FC between the dACC and two clusters were positively correlated with conformity propensity (voxel-level *p* < 0.002, cluster-level *p* < 0.05, corrected, Figure 3F), including the bilateral PO (right, 241 voxels, MNI coordinates: 58, −21, 20; left, 463 voxels, MNI coordinates: −55, −21, 10). In addition, the correlations remained unchanged while controlling for the participants’ head motion and gender (dACC-rPO: partial *r* = 0.210, *p* = 0.001; dACC-lPO: partial *r* = 0.262, *p* < 0.001). Taken together, these results suggest that from the right thalamus to the bilateral insula to the PG to the dACC, these regions might not only play parallel hub-like roles in punishment sensitivity but also seem to have integrated as an ascending pathway to facilitate conformity behaviors (Figure 4).

## Discussion

Using rs-fMRI, we demonstrated that task-independent FC in the PN related to individual differences in conformity propensity: higher WNC in the PN was associated with a stronger tendency to conform. Specifically, first, we identified the right thalamus, bilateral insula, PG, and dACC as hubs for integrating all other regions in the PN. Then, the correlation analysis between WNC and the conformity scores demonstrated that individuals with stronger WNC in the right thalamus, left insula, PG, and dACC (i.e., high PN integration) exhibited considerably higher conformity propensity. Furthermore, through seed-based analysis, the results suggested that the specific connections from all four brain regions seemed to form an ascending pathway and that the connection of each phase in this pathway all contribute to conformity propensity. Therefore, stronger WNC in the PN might predispose conformity behaviors by fostering punishment sensitivity. Every phase in this ascending pathway for punishment sensitivity was positively correlated with conformity propensity.

By using the GBC approach, our finding that the right thalamus, left insula, PG, and dACC are hub areas integrating the whole PN is consistent with previous findings that these brain regions play a central role in punishment processing, such as the processing of aversive, and painful stimuli (Frot et al., 2007; Chai et al., 2010; Straube and Miltner, 2011; Kobayashi, 2012; Wiech et al., 2014). Thus, the fact that these four hubs integrating the PN at resting state indicate that they were in an ongoing readiness state for priming of multiple potential punishments (Simmons and Martin, 2011; Shen, 2015). This readiness state positively correlated with individuals’ conformity propensity, suggesting that this task-independent neural functioning might predispose individuals toward conformity.

Also, seed-based FC-conformity correlation analyses were conducted with four clusters as seeds in the right thalamus, left insula, PG, and dACC, respectively, and the results showed that the FCs in the four hub regions seemed to form an ascending pathway that was positively correlated with conformity propensity in each phase. It is also worth mentioning that the PO was not the hubs in the PN but connected with two hubs (i.e., the PG and dACC) in the ascending pathway to contribute to the behavior tendency of conformity. This result is consistent with previous studies that have indicated that the function of the PO might not be the hub region to integrate the whole PN, but that it plays an essential role in transmitting signals within the hubs (Eickhoff et al., 2010; Garcia-Larrea, 2012; Mano et al., 2017). More importantly, unilateral severe physical punishment (e.g., painful stimulation) evoked bilateral activation of PO but also activated the insula, PG, and cingulate cortices in the contralateral hemisphere in completely callosotomized patients (Fabri et al., 2002), which indicated that PO could play a powerful function in transferring information bilaterally, even in subjects with resection of the corpus callosum and distributing signals to both hemispheric brain regions. Thus, the major role of the PO could be facilitating connectivity within the PG and dACC in the ascending pathway. Our results suggested that the mechanism of punishment sensitivity comprises multiple phases of processing (Ernst et al., 2006), and this sensitivity could result from stronger connectivity of one or multiple phases.

Specifically, this punishment sensitivity pathway in the PN could be divided into three phases (Figure 4): Phase 1 is the “input phase” and refers to the connectivity from the thalamus to the insula, which is responsible for processing early sensory input (Dum et al., 2009; Liang et al., 2012; Cho et al., 2013). Phase 2 is the “affective and sensory processing phase” and contributes to the connection between the insula, PO, and PG (where the somatosensory cortex is located). According to research, the insula is responsible for processing the “affective” components of punishment stimulus (Touroutoglou et al., 2012; Duerden et al., 2013; Rogers-Carter et al., 2018), activation during aversive anticipation (Simmons et al., 2006; Carlson and Mujica-Parodi, 2010; Haase et al., 2014), and arousal during negative affection processing (Caria et al., 2010; Duerden et al., 2013), whereas the somatosensory cortex is responsible for processing “sensory-discrimination” and is implicated in self-awareness of a person’s own body (Frot et al., 2007; Khalsa et al., 2009) as the perception of bodily states playing a crucial role for affective and emotional experiences (Straube and Miltner, 2011). Phase 3 occurs in the ascending pathway and is the combined affective and sensory signal projected to the dACC (i.e., accomplished by the connectivity within the somatosensory cortex, PO, and dACC), which acts as a neural “alarm system” or conflict monitor, detecting “something is wrong” and preparing for a response (Eisenberger and Lieberman, 2004; Ullsperger et al., 2004; Gonzalez et al., 2014; Chester and DeWall, 2015; Coste and Kleinschmidt, 2016). The connectivity in every phase is positively associated with conformity propensity, which means that every phase is preparing for punishment stimulus and the sensitivity in each phase plays a role in explaining individual differences in conforming tendency.

Also, two regions included in our predefined PN did not seem to play essential roles in punishment sensitivity and did not contribute to conformity propensity. First, the dorsal striatum is mentioned in a few previous studies to be associated with punishment avoidance (Seymour et al., 2007; Delgado et al., 2008; Pauli et al., 2015). While reward processing being associated with striatum activation has been almost consistently reported, results regarding punishment processing have been less consistent (Rutledge et al., 2009; Jocham et al., 2011; Eisenegger et al., 2014). The fact is that the meaning of positive or negative outcomes always been reframed in studies about reward- or punishment-processing tasks, which means the absence of punishment could be perceived as a reward (Vlaev et al., 2011; Rangel and Clithero, 2012; Palminteri et al., 2015). Thus, the dorsal striatum might play a major role in value-coding in these tasks, rather than being a response specific to punishment. Second, the amygdala has not presented as a hub area in punishment sensitivity. The possible reason is that the amygdala is an area associated with emotional processing, such as emotional salience, valence, and discrimination (Pessoa and Adolphs, 2010). So in punishment relevance tasks, the function of the amygdala is more likely to process the emotional response to individuals’ own errors before punishment is inflicted (Jackson et al., 2015) or to the succeeding emotional processing after suffering punishment (Sladky et al., 2013). Thus, it is not responsible for processing the direct experience associated with the punishment itself; therefore, it has not played a role in punishment sensitivity or contributed to conformity propensity in this study.

## Limitations and Future Directions

The limitations and several unaddressed issues of the present study need to be explored in future research. First, given that the processing ascending pathway in the PN presented in this study was based on seed-based analysis, future studies are invited to investigate directed FC within PN (e.g., using Granger causality analysis, GCA; Khazaee et al., 2017; Price et al., 2017; Xue et al., 2019), and how the directed FC in the PN are associated with conformity propensity. Second, the present study used resting-state FC when participants were not performing punishment processing tasks since the present study aims to investigate the intrinsic FC in the PN as an indicator for task-free, stable trait-like neural activity in potential punishment (Tavor et al., 2016), yet future fMRI studies measuring task-state FC during the performance of the punishment processing relevant tasks (Palminteri et al., 2012; Palminteri and Pessiglione, 2017) may help further elucidate the distinct function of each region related to punishment processing and the specific role they play in promoting conformity behaviors. Third, the generalization of the present finding is limited by the purely college-aged sample. It will be interesting for future studies to investigate the punishment sensitivity at the neural level in other age groups and the corresponding associations with group varieties of conformity propensity.

## Conclusion

In summary, the present study evidences that a neural trait marker—task-independent FC in the PN—explains individual differences in conformity propensity. That might be the reason why the conformity phenomenon is so prevalent in our society today because the neural connectivity in the PN is a consistent and automatic motivational factor in our brain. Hence, our study revealed a paradox: we conform because of sensitivity to punishment, but avoiding potential punishment leads us to be a “one-dimensional man,” which itself is the most severe punishment. The significance of this study is probably cautionary at best. As American historian Wilfred M. McClay evaluated Riesman’s *The Lonely Crowd*, “It warns us against the peculiar forms of bondage to which our era is especially prone. And in doing so, it draws us into a deeper consideration of what freedom might be, both now and in the future,” (McClay, 1998). Thus, constructing a new sensibility of being nonconforming is the antidote to regaining one’s drive for personal liberation.

## Data Availability Statement

The raw data supporting the conclusions of this article will be made available by the authors, without undue reservation.

## Ethics Statement

The studies involving human participants and all investigation protocols were reviewed and approved by the Institutional Review Board of Beijing Normal University. The patients/participants provided their written informed consent to participate in this study.

## Author Contributions

YD contributed to the study conception and design, performed data analysis and interpretation, and wrote the manuscript. YW contributed to the study conception and design, and critically revised the article. MY and XT performed material preparation and data collection. JL designed the work and critically revised the article. All authors contributed to the article and approved the submitted version.

## Conflict of Interest

The authors declare that the research was conducted in the absence of any commercial or financial relationships that could be construed as a potential conflict of interest.

## Footnotes

^1^https://github.com/neurosynth/

^2^http://fsl.fmrib.ox.ac.uk/fsl/fslwiki/

^3^http://afni.nimh.nih.gov
